# Oxidative stress regulates glycogen synthase kinase-3 in lymphocytes of diabetes mellitus patients complicated with cerebral infarction

**DOI:** 10.1515/med-2024-1095

**Published:** 2024-11-29

**Authors:** Man Wang, Ying Qu, Shujin Wang, Zhongsen Qu

**Affiliations:** Department of Rehabilitation, Shanghai Sixth People’s Hospital Affiliated to Shanghai Jiao Tong University School of Medicine, Shanghai, 200233, China; Department of Neurology, Kunming Medical University, Kunming 650500, China; Department of Neurology, The First Hospital of Zibo Affiliated to Weifang Medical University, Zibo, 25520, China

**Keywords:** diabetes mellitus, cerebral infarction, oxidative stress, glycogen synthase kinase-3

## Abstract

**Objective:**

To explore the role of oxidative stress on glycogen synthase kinase-3 in lymphocytes of diabetes mellitus (DM) patients complicated with cerebral infarction (CI).

**Materials and methods:**

A total of 186 DM patients were enrolled according to the inclusion criteria and exclusion criteria, including 89 DM patients alone (DM group) and 97 DM patients with CI (DM + CI) group. Eighty-one patients with CI were selected as the CI group, and 80 normal subjects over 50 years were selected as the control group. Superoxide dismutase (SOD) activity, glutathione peroxidase (GSH-Px) activity, and malondialdehyde (MDA) content in serum were determined by colorimetric assays. Phosphorylation of GSK-3β was measured by enzyme-linked immunosorbent assay.

**Results:**

(1) Compared with the control group, the SOD and GSH-Px activities in the DM group and DM + CI group were decreased, accompanied by higher MDA content. Furthermore, phosphorylation of GSK-3β was decreased. (2) In the DM + CI group, SOD activity was decreased on days 7 and 10 and month 3 compared to the CI group and was decreased on day 7 compared to the DM group. MDA content was increased from day 0 to month 3 compared to the CI group. On days 1, 7, and 10, GSH-Px activity was lower than the DM group, and on day 10 and month 3, it was lower than the CI group. Phosphorylation of GSK-3β was decreased on days 7 and 10 compared to the DM group and was decreased from day 1 to month 3 compared to the CI group.

**Conclusion:**

In the present study, we demonstrated that the oxidative stress in peripheral lymphocytes of DM patients complicated with CI was stronger, and the GSK-3 activity was higher. It suggested that oxidative stress might enhance the GSK-3 activity, which might provide a diagnostic and therapeutic approach for DM complicated with CI, and targeting GSK-3 is a promising therapeutic target for the treatment of type 2 diabetes and its complications.

## 1 Introduction

Diabetes mellitus (DM) and cerebral infarction (CI) are two major diseases increasing with aging that affect human health, and DM can cause various complications, including macrovascular and microvascular lesions, which is an independent risk factor for CI [1]. Oxidative stress was found to be the cause of DM and its complications [2]. In addition, chronic hyperglycemia and insulin resistance in diabetes are also associated with oxidative stress [3,4,5]. In diabetic patients, vascular endothelial cells are damaged by oxidative stress, which increases intravascular permeability and ultimately contributes to the development of macrovascular and microvascular diseases. Various mechanisms are involved in the pathogenesis of CI, among which oxidative stress injury and apoptosis seem to be the most critical events [6]. Oxidative stress can lead to increased reactive oxygen species generation and mitochondrial dysfunction in the brain, protein, and DNA damage in ischemic neurons, even neuronal cell apoptosis and death, and eventually brain injury after reperfusion [7].

Various studies have shown that the total antioxidant status of T2DM is significantly reduced, manifested by lower levels of antioxidant enzymes such as glutathione peroxidase (GSH-Px), catalase, and superoxide disoxidase and considerably higher levels of peroxide and other biomarkers of oxidative stress [7]. And most previous studies compared healthy individuals with T2DM patients and showed that superoxide dismutase (SOD) activity and GSH-Px activity in T2DM patients were decreased, while malondialdehyde (MDA) was increased [8].

It is well known that insulin regulation on glucose (GS) metabolism is highly dependent on the PI3K/AKT signaling pathway [9]. In the presence of insulin stimulation, the PI3K-Akt signaling pathway is initiated, inducing the phosphorylation of GSK3, thereby stimulating glycogen synthesis [10]. In addition, the PI3K/Akt pathway is one of the important signaling pathways that can regulate neural cell survival and death, neural cell proliferation, and synaptic plasticity, and its activation significantly reduces brain injury and protects brain neurons from hypoxia/reoxygenation-induced apoptosis [6,11,12]. It is suggested that the PI3K/Akt signaling pathway is an important target for the treatment of ischemic stroke [13]. Therefore, activation of the PI3K-Akt signaling pathway may play a role in CI and diabetes. GSK-3 is a downstream kinase of the PI3K/AKT signaling pathway and can regulate the signaling pathways involved in the pathogenesis of ischemic stroke. Increasing evidence suggests that GSK 3 inhibition can reduce cerebral ischemia/reperfusion injury [14].

Research has confirmed that lymphocytes can easily pass through the blood–brain barrier and reflect brain changes [15]. Besides, studies have investigated the damage to cellular DNA caused by oxidative stress of peripheral lymphocytes in patients with DM [8]. It has not been clear whether changes in oxidative stress of peripheral lymphocytes affect GSK-3 in DM patients with CI. Therefore, in this study, we examined the changes in oxidative stress and GSK-3β activity in peripheral lymphocytes to explore their possible roles in DM patients complicated with CI.

## 2 Methods

### 2.1 Study design and patient selection

A total of 186 newly admitted T2DM subjects who were admitted to the inpatient wards and outpatient clinics between December 2019 and December 2020 were enrolled in this study. The patients with T2DM were divided into those with CI (DM + CI group, *N* = 97) and those without any clinical signs and symptoms of CI (DM group, *N* = 89) according to the inclusion criteria and exclusion criteria. Furthermore, 81 patients with CI group who were admitted to the hospital within the first 24 h of symptom onset were selected.

Inclusion criteria for the study were: Patients with DM met the diagnostic criteria for DM from the American Diabetes Association in 2017 [16], and their diabetes was type II DM.

Patients with CI met the diagnostic criteria for CI from the American Heart Association/the American Stroke Association in 2018 [17], and their CI was confirmed by MRI or CT.

Exclusion criteria for the study were intracranial infection, subarachnoid hemorrhage, or intracerebral hemorrhage; malignancy; history of hematological system diseases and the tendency for bleeding; history of rheumatic heart disease and atrial fibrillation; history of liver and respiratory disease; history of surgery and trauma (1 month before the experiment); and history of autoimmune disease or any treatment with immunosuppressive agents. Patients requiring thrombolytic therapy.

Eighty normal subjects aged ≥50 years in the medical examination center in the same period were recruited as healthy controls without any treatment. All subjects were in general good health and had normal blood pressure, lipids, blood GS, liver, kidney, and cardiopulmonary function.

The gender and the age of the patients were not significant (*P* > 0.05).

### 2.2 Treatments

#### 2.2.1 Treatments for DM

Metformin hydrochloride tablets (Beijing Jingfeng Pharmaceutical Co., Ltd, approved number is H11021518), 0.25 g orally three times a day, plus gliclazide tablets (Tianjin Junan Biopharmaceutical Co., Ltd, approval number is H20056883) 30 mg, once a day. Controlling the risk factors. Diet guidance, exercise guidance, and health education were provided during the treatment period.

#### 2.2.2 Treatments of CI

All patients were given standard therapy, including controlling blood pressure, maintaining the water, electrolyte, and acid–base balance, improving brain cell metabolism, reducing cerebral edema, regulating blood lipid, and other treatments. At the same time, aspirin (Bayer Medical Care Ltd. approval number is J20171021), 100 mg, once a day.

### 2.3 Methods

#### 2.3.1 Sample preparation

We collected the blood on day 0, day 1, day 7, day 10, month 1, and month 3. All subjects were drawn in the early morning after 12 h of fasting. Venous blood (2 ml) was drawn from each subject with procoagulant tubes produced by BD company. The samples were centrifuged at 1,500 *g* for 10 min at 4°C. Serum was separated from blood and stored at −80°C.

#### 2.3.2 Measurements of SOD, MDA, and GSH-Px in serum

The serum samples stored at −80°C were taken. MDA content and SOD activity were measured using ultraviolet spectrophotometry. SOD activity was determined by the xanthine oxidase method, the kit was provided by Nanjing Jiancheng Bioengineering Institute (A001-3-2), and MDA content was measured using the thiobarbituric acid method, the kit was provided by Nanjing Jiancheng Bioengineering Institute (A001-3-2). The GSH-Px activity was determined by the dithiobis nitrobenzoic acid method, the kit was provided by Nanjing Jiancheng Bioengineering Institute (A005-1-2).

#### 2.3.3 Lymphocyte samples

Fasting venous blood (10 ml) was collected from subjects of different groups in the heparinized tube and was diluted with an equal amount of PBS. The diluted blood sample (6 ml) was added to a centrifuge tube (15 ml), and then lymphocyte separation fluid (3 ml) was added to the tube. The sample was centrifuged at 800*g* for 20 min at room temperature, and lymphocytes were collected from the interface and transferred to another centrifuge tube. Then, the sample was diluted with PBS and centrifuged at 800*g* for 20 min, and the supernatant was discarded. The precipitates were washed three times with PBS and diluted into a suspension of 4–10 × 10^6^ with PBS. 100 µl cell lysates were added, and the cells were then disrupted by sonication (10 s, 3 times) and centrifuged at 1,200*g* for 20 min. The supernatant was taken, and protein concentration was determined by bicinchoninic acid assay and stored at −80°C.

#### 2.3.4 Measurement of the phosphorylation of GSK-3βin lymphocytes

Samples stored were taken, and the phosphorylation of GSK-3β was determined using enzyme-linked immunosorbent assay. The kit was provided by Shanghai Hushang Biotechnology (2435-1). The absorbance value (*A* value) was detected at 450 nm wavelength by a microplate reader, and the *A* values obtained from the standard product were used to plot a standard curve.

### 2.4 Statistical analysis

All data were analyzed using SPSS version 13.0. Data were tested for normality using the Shapiro-Wilk test. Homogeneity of variance was tested by Levene’s. The results were expressed as means ± standard deviation (*x* ± *s*), and normally distributed variables were analyzed using a one-way repeated-measures analysis of variance. Non-normally distributed data were analyzed using the Wilcoxon test. Differences between the two groups were evaluated statistically by use of the least significant difference; the difference was considered statistically significant when *P* < 0.05.


**Ethical approval:** The study was approved by the Ethics Committee of The First Hospital of Zibo (YXLL2019072751).
**Informed consent:** Informed consent was obtained from the patient or relatives when the patient was not able to give informed consent.

## 3 Results

### 3.1 Baseline characteristics

As shown in Table 1, baseline characteristics showed statistical differences among groups. Specifically, in the DM group, blood GS, total cholesterol (TC), and low-density lipoprotein cholesterol (LDL-C) were higher than those in the control group (*P* < 0.05, *P* < 0.05, *P* < 0.05), while blood pressure and triglyceride (TG) showed no difference (*P* > 0.05, *P* > 0.05). The blood pressure and lipids in the CI group were significantly higher than in controls (*P* < 0.05, *P* < 0.05). The DM + CI group had higher blood GS, lipids, and blood pressure than controls (*P* < 0.05, *P* < 0.05, *P* < 0.05), higher LDL cholesterol than the DM group and CI group (*P* < 0.05, *P* < 0.05).

**Table 1:** Biochemical indicators of the patients among the groups

Group	*N*	Sex (female/male, *n*)	Age (years)	GS (mmol/L)	TC (mmol/L)	TG (mmol/L)	C- LDL (mmol/L)	SBP (mmHg)	DBP (mmHg)
Control group	80	45/35	59.4 ± 6.6	5.13 ± 0.61	4.13 ± 0.82	1.58 ± 0.32	2.48 ± 0.24	128.5 ± 14.8	76.9 ± 9.6
DM group	89	42/47	63.2 ± 7.5	11.87 ± 2.36^*^	7.24 ± 1.46^*^	1.95 ± 0.49	5.15 ± 0.94^*^	137.4 ± 16.2	87.2 ± 9.3
CI group	81	43/38	67.8 ± 8.9	5.78 ± 0.92	7.94 ± 1.37^*^	2.96 ± 0.62^*^	5.48 ± 1.54^*^	159.7 ± 24.2^*^	93.5 ± 11.2^*^
DM + CI group	97	54/43	62.9 ± 6.8	14.65 ± 2.47^**^	9.49 ± 2.78^**^	3.23 ± 0.86^*^	9.86 ± 2.73^**Δ#^	166.8 ± 22.9^*^	103.5 ± 13.4^**^
*F*/*χ* ^*2*^		9.745	6.987	2.254	2.216	2.477	0.986	1.184	0.759
*P*		0.781	0.603	0.036	0.031	0.042	0.008	0.012	0.000

Note: ^*^For comparison between the control group and the other groups, *P* < 0.05, ^**^indicates *P* < 0.01; ^Δ^for comparison between the DM group and the other groups, *P* < 0.05; ^#^for comparison between the CI group and the other groups, *P* < 0.05.

### 3.2 Oxidative stress detection

**Figure 1:**
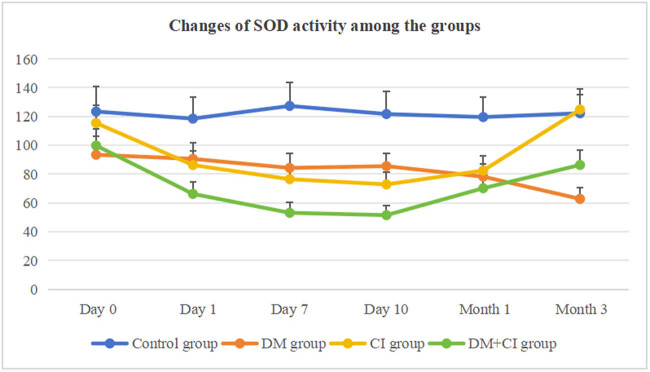
SOD activity among the groups.

**Figure 2:**
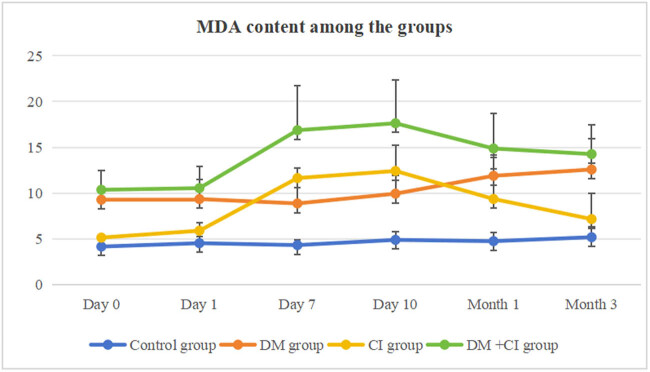
MDA content among the groups.

Tables 2 and 3 show the oxidative stress parameters among the groups. Figures 1 and 2 show the changes in the oxidative stress biomarkers among the groups. Table 2 shows the differences in SOD activity among different groups. Figure 1 shows the changes in SOD activity among the groups. In the DM group, SOD activity was decreased from day 0 to month 3 compared with the control group (*P* < 0.05, *P* < 0.05, *P* < 0.05, *P* < 0.05, *P* < 0.01, *P* < 0.01). SOD activity in the CI group was decreased from day 1 to month 1 compared to the control group (*P* < 0.05, *P* < 0.01, *P* < 0.01, *P* < 0.01). Compared to day 0, SOD activity was decreased from day 1 to month 1(*P* < 0.05, *P* < 0.01, *P* < 0.01, *P* < 0.05), and it returned to normal by month 3 (*P* > 0.05). SOD activity in the DM + CI group was decreased from day 0 to month 3 compared to the control group (*P* < 0.05, *P* < 0.01, *P* < 0.01, *P* < 0.01, *P* < 0.01, *P* < 0.01). SOD activity was decreased on day 7, 10, and month 3 compared to the CI group (*P* < 0.05, *P* < 0.05, *P* < 0.01) and was lower on day 7 compared to the DM group (*P* < 0.05).

**Table 2:** Changes of SOD activity (ng/ml) among the groups

Group	*N*	Day 0	Day 1	Day 7	Day 10	Month 1	Month 3
Control group	80	123.45 ± 17.43	118.54 ± 14.71	127.36 ± 16.35	121.73 ± 15.96	119.62 ± 13.75	122.26 ± 12.66
DM group	89	93.47 ± 12.76^*^	90.58 ± 11.36^*^	84.32 ± 9.87^*★^	85.56 ± 8.66^*^	78.25 ± 8.72^**^	62.84 ± 7.81^**★★^
CI group	81	115.48 ± 12.33	86.32 ± 9.56^*#^	76.51 ± 8.12^**##^	72.89 ± 8.34^**##^	82.52 ± 10.14^**#^	124.83 ± 14.39
DM + CI group	97	99.85 ± 11.81^*^	66.31 ± 8.56^**#^	53.18 ± 7.15^**△##★^	51.54 ± 6.92^**##★^	70.25 ± 8.94^**#^	86.37 ± 10.28^**★★^
*F*, *P*		5.634, 0.042	6.457, 0.032	8.798, 0.030	8.666, 0.028	9.142, 0.000	9.886, 0.000
Compare within groups							
Time	*N*	*F, P*	Day 1 vs Day 0, LSD-t, *P*	Day 7 vs Day 0, LSD-t, *P*	Day 10 vs Day 0, LSD-t, *P*	Month 1 vs Day 0, LSD-t, *P*	Month 3 vs Day 0, LSD-t, *P*
DM group	89	4.124, 0.300					
CI group	81	12.131, 0.030	6.908, 0.026	10.722, 0.005	10.926, 0.003	7.223, 0.025	2.980, 0.360
DM + CI group	97	13.482, 0.036	8.844, 0.018	12.382, 0.002	5.856, 0.024	4.833, 0.030	2.567, 0.235

Note: ^*^For comparison between control group and the other groups, *P* < 0.05, ^**^indicates *P* < 0.01; ^**△**^for comparison between DM group and the other groups, *P* < 0.05; ^★^for comparison between CI group and the other groups, *P* < 0.05, ^★★^indicates *P* < 0.01; ^#^for comparison between day 0 and other time spot, *P <* 0.05, ^##^indicates *P* < 0.01.

**Table 3:** MDA content among the groups (nmol/ml) ( \[\overline{X}\pm S]\] )

Group	*N*	Day 0	Day 1	Day 7	Day 10	Month 1	Month 3
Control group	80	4.16 ± 0.69	4.53 ± 0.74	4.32 ± 0.58	4.89 ± 0.91	4.75 ± 0.98	5.18 ± 1.12
DM group	89	9. 28 ± 2.16^*★^	9.35 ± 2.49^*★^	8.78 ± 1.96^*^	9.94 ± 2.27^*^	11.89 ± 3.34^**^	12.58 ± 3.77^**★^
CI group	81	5.14 ± 0.87	5.89 ± 1.12	11.64 ± 2.83^**#^	12.42 ± 3.27^**##^	9.36 ± 2.78^*^	7.16 ± 1.28
DM + CI group	97	10.36 ± 2.12^*★^	10.54 ± 2.36^*★^	16.87 ± 4.84^**★△△#^	17.63 ± 4.72^**★△△##^	14.87 ± 3.82^**★**△**^	14.26 ± 3.23^**★★^
*F*, *P*		5.264, 0.040	5.456, 0.042	8.988, 0.008	9.065, 0.008	9.821, 0.000	9.862, 0.000
Compare within groups							
time	*N*	*F, P*	Day 1 vs Day 0, LSD-t, *P*	Day 7 vs Day 0, LSD-t, *P*	Day 10 vs Day 0, LSD-t, *P*	Month 1 vs Day 0, LSD-t, *P*	Month 3 vs Day 0, LSD-t, *P*
DM group	89	1.648, 0.320					
CI group	81	7.512, 0.025	0.523, 0.556	3.252, 0.042	5.840, 0.004	0.764, 0.064	2.500,0.826
DM + CI group	97	8.250, 0.020	0.844, 0.980	7.382, 0.028	8.560, 0.003	1.432, 0.763	1.065,0.854

Note: ^*^for comparison between control group and the other groups, *P* < 0.05, ^**^indicates *P* < 0.01; ^**△**^for comparison between DM group and the other groups, *P* < 0.05; ^★^for comparison between CI group and the other groups, *P* < 0.05, ^★★^indicates *P* < 0.01; ^#^for comparison between day 0 and other time spot, *P* < 0.05, ^##^indicates *P* < 0.01.

Table 3 and Figure 2 show the changes in MDA content among different groups. MDA content of the DM group was increased from day 0 to month 3 compared to the control group (*P* < 0.05, *P* < 0.05, *P* < 0.05, *P* < 0.05, *P* < 0.01, *P* < 0.01). In the CI group, MDA content was increased from day 7 to month 1 compared to the control group (*P* < 0.01, *P* < 0.01, *P* < 0.05). Compared to day 0, MDA content was increased on day 7 and day 10 (*P* < 0.05, *P* < 0.01). In the DM + CI group, MDA content was increased from day 0 to month 3 compared to the control group (*P* < 0.05, *P* < 0.05, *P* < 0.01, *P* < 0.01, *P* < 0.01, *P* < 0.01). Compared to the DM group, MDA content was increased on day 7, day 10, and month 1 (*P* < 0.01, *P* < 0.01, *P* < 0.05). MDA content of the DM + CI group was increased from day 0 to month 3 compared to the CI group (*P* < 0.05, *P* < 0.05, *P* < 0.05, *P* < 0.05, *P* < 0.05, *P* < 0.01).

**Figure 3:**
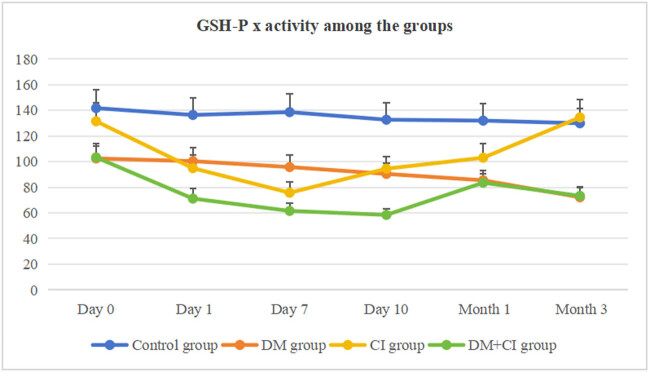
GSH-Px activity among the groups.

Table 4 and Figure 3 show changes in the GSH-Px activity among different groups. The GSH-Px activity in the DM group was decreased from day 0 to month 3 compared to the control group (*P* < 0.05, *P* < 0.05, *P* < 0.05, *P* < 0.05, *P* < 0.01, *P* < 0.01). In the CI group, the GSH-Px activity was decreased from day 1 to month 1 compared to the control group (*P* < 0.05, *P* < 0.01, *P* < 0.05, *P* < 0.05). GSH-Px activity was decreased from day 1 to month 1 compared to day 0 (*P* < 0.05, *P* < 0.01, *P* < 0.05, *P* < 0.05), and it returned to normal by month 3 (*P* > 0.05). In the DM + CI group, the GSH-Px activity was decreased from day 0 to month 3 compared to the control group (*P* < 0.05, *P* < 0.01, *P* < 0.01, *P* < 0.01, *P* < 0.01, *P* < 0.01). On day 1,7, 10, GSH-Px activity was lower than in the DM group (*P* < 0.05, *P* < 0.05, *P* < 0.05), and on day 10 and the month 3, it was lower than that in the CI group (*P* < 0.05, *P* < 0.01).

**Table 4:** GSH-Px activity among the groups (U/ml)

Group	*N*	Day 0	Day 1	Day 7	Day 10	Month 1	Month 3
Control group	80	141.65 ± 14.47	136.31 ± 13.22	138.52 ± 14.38	132.62 ± 13.42	131.91 ± 13.29	129.85 ± 11.48
DM group	89	102.44 ± 9.43^*^	100.32 ± 10.26^*^	95.73 ± 9.38^*^	90.40 ± 8.65^*^	85.42 ± 7.86^**★^	72.21 ± 7.51^**★★#^
CI group	81	131.4 ± 14.11	94.84 ± 10.25^*#^	75.72 ± 8.55^**##^	94.46 ± 9.49^*#^	102.98 ± 11.30^*#^	134.42 ± 13.94
DM + CI group	97	103.23 ± 10.72^*^	71.18 ± 7.72^**#△^	61.57 ± 6.23^**#△^	58.46 ± 4.82^**##△★^	83.53 ± 7.04^**^	73.29 ± 7.21^**★★#^
*F*, *P*		8.342, 0.022	9.578, 0.020	12.980, 0.000	12.660, 0.000	11.422, 0.000	10.688, 0.000
Compare within groups							
Time	*N*	*F, P*	Day 1 vs Day 0, LSD-t, *P*	Day 7 vs Day 0, LSD-t, *P*	Day 10 vs Day 0, LSD-t, *P*	Month 1 vs Day 0, LSD-t, *P*	Month 3 vs Day 0, LSD-t, *P*
DM group	89	5.428, 0.046	1.342,0.345	1.087,0.476	0.782,0.765	0.988,0.566	1.332,0.040
CI group	81	12.131, 0.030	9.800, 0.018	10.242, 0.002	8.260, 0.032	6.232, 0.042	5.980,0.630
DM + CI group	97	13.482, 0.036	9.440, 0.025	10.082 0.030	15.560, 0.004	4.330, 0.038	4.675,0.350

Note: ^*^for comparison between control group and the other groups, *P* < 0.05, ^**^indicates *P* < 0.01; ^**△**^for comparison between DM group and the other groups, *P* < 0.05; ^★^for comparison between CI group and the other groups, *P* < 0.05, ^★★^indicates *P* < 0.01; ^#^for comparison between day 0 and other time spot, *P* < 0.05, ^##^indicates *P* < 0.01.

### 3.3 Phosphorylation of GSK-3β among different groups

**Figure 4:**
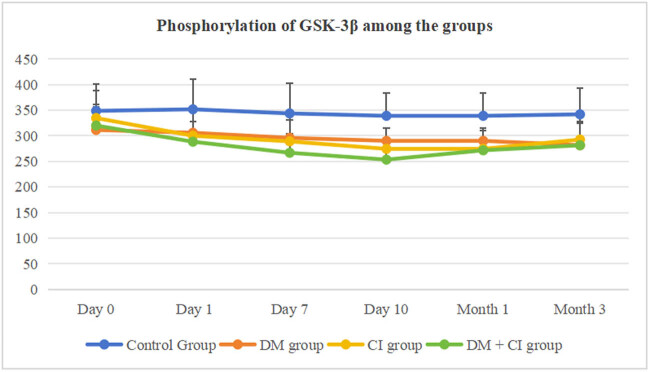
Phosphorylation of GSK-3β among the groups.

As shown in Table 5 and Figure 4, phosphorylation of GSK-3β in the DM group was decreased from day 0 to month 3 compared to the control group (*P* < 0.05, *P* < 0.05, *P* < 0.05, *P* < 0.05, *P* < 0.05, *P* < 0.01). Compared to day 0, phosphorylation of GSK-3β was decreased in month 3 (*P* < 0.05). In the CI group, phosphorylation of GSK-3β was decreased from day 1 to month 1 compared to the control group (*P* < 0.05, *P* < 0.01, *P* < 0.01, *P* < 0.01, *P* < 0.05), and it returned to normal by month 3 (*P* > 0.05). In the DM + CI group, phosphorylation of GSK-3β was decreased from day 0 to month 3 compared to the control group (*P* < 0.05, *P* < 0.01, *P* < 0.01, *P* < 0.01, *P* < 0.01, *P* < 0.01). Compared to day 0, phosphorylation of GSK-3β was decreased from day 0 to month 3 (*P* < 0.05, *P* < 0.05, *P* < 0.05, *P* < 0.05, *P* < 0.05). Compared to the DM group, phosphorylation of GSK-3β was decreased on days 7 and 10 (*P* < 0.05, *P* < 0.05) and was decreased from day 1 to month 3 compared to the CI group (*P* < 0.05, *P* < 0.01, *P* < 0.01, *P* < 0.01, *P* < 0.01).

**Table 5:** Phosphorylation of GSK-3β among the groups (pg/ml) ( \[\overline{X}\pm S]\] )

Group	*N*	Day 0	Day 1	Day 7	Day 10	Month 1	Month 3
Control Group	80	348.56 ± 52.38	351.58 ± 58.48	343.54 ± 58.32	338.86 ± 44.95	338. 86 ± 44.95	341.78 ± 51.29
DM group	89	311.26 ± 45.65^*^	305.81 ± 44.45^*^	295.78 ± 48.59^*^	290.04 ± 42. 52^*^	290.04 ± 42. 52^*^	281.51 ± 45.37^★** #^
CI group	81	334.56 ± 54.39	299.92 ± 47.72^*★^	288.96 ± 41.46^**★★^	274.36 ± 39.99^**★★^	274.36 ± 39.99^**★★^	292.53 ± 46.23^*★^
DM + CI group	97	319. 88 ± 40.52^*^	288.34 ± 38.67^**#★^	266.73 ± 36.43^**#△★★^	253.34 ± 32.28^**#△★★^	271.55 ± 39.08^**#★★^	281.34 ± 42.48^**#★★^
*F*, *P*		5.429, 0.042	8.785, 0.024	9.988, 0.002	10.360, 0.000	10.425, 0.000	10.680, 0.000
Compare within groups							
Time	*N*	*F, P*	Day 1 vs Day 0, LSD-t, *P*	Day 7 vs Day 0, LSD-t, *P*	Day 10 vs Day 0, LSD-t, *P*	Month 1 vs Day 0, LSD-t, *P*	Month 3 vs Day 0,LSD-t, *P*
DM group	89	5.428, 0.006	1.320,0.368	1.788,0.465	1.859,0.708	1.998,0.516	2.332,0.046
CI group	81	1.310, 0.006	2.542,0.030	2.732,0.033	2.783,0.325	3.296,0.024	3.533,0.020
DM + CI group	97	6.822, 0.030	5.440, 0.020	6.008, 0.030	6.560, 0.024	6.336, 0.028	6.674, 0.030

Note: ^*^for comparison between control group and the other groups, *P* < 0.05, ^**^indicates *P* < 0.01; ^**△**^for comparison between DM group and the other groups, *P* < 0.05; ^★^for comparison between CI group and the other groups, *P* < 0.05, ^★★^indicates *P* < 0.01; ^#^for comparison between day 0 and other time spot, *P* < 0.05, ^##^indicates *P* < 0.01.

## 4 Discussion

This study investigated the role of oxidative stress on glycogen synthase kinase-3 in lymphocytes of DM patients complicated with CI. The results showed that the oxidative stress biomarkers including SOD, GSH-Px, and MDA were changed in DM patients complicated with CI. Besides, GSK-3β activity in DM patients complicated with CI was increased. The findings of the study suggested that oxidative stress can modulate GSK-3β activity in peripheral lymphocytes of DM patients complicated with CI.

Increasing studies have suggested that oxidative stress plays a pivotal role in the pathogenesis and progression of diabetes [2]. Oxidative stress is that reactive oxygen species will increase when the body receives harmful stimuli, leading to the imbalance between the level of reactive oxygen species and antioxidant capacity, and resulting in tissue damage [1]. Most biologic cells have endogenous antioxidant systems including various enzymes like SOD and GSH that protect cells against free radicals attacking [18,19]. SOD and GSH are mainly used for scavenging harmful metabolic substances and reflecting the antioxidant capacity of the human body [7]. Lipid peroxidation is a major causative factor for the development of oxidative stress that leads to overt T2DM and its associated micro- and macro-vascular complications [20]. MDA, the terminal product of lipid peroxidation, can reflect the content of reactive oxygen species and the degree of oxidative damage. The higher the MDA, the more serious the free radical damage is [21]. These oxidation products can be used as biological markers to assess the level of oxidative stress or reactive oxygen species in the body. Higher blood GS not only affects the activity of various antioxidant enzymes but also glycosylates non-proteases and increases the level of oxygen free radicals [1]. Previous studies have reported that SOD and GSH-Px activity were decreased in T2DM patients, while MDA content was increased [8,22]. This study confirmed that SOD and GSH-Px activity in peripheral lymphocytes of DM patients were decreased, and MDA content was increased that consistently persisted until 3 months after diabetes. It proved that oxidative stress was involved in the course of diabetes. Measurement of oxidative stress biomarkers may be one of the optional tools for the diagnosis and prediction of type 2 diabetes.

It is generally recognized that T2DM-associated vascular disease is mainly associated with vascular endothelial injury and endothelial dysfunction, which are the initial stage of vasculopathy and a vital prognostic indicator of diabetic vascular complications [23]. In diabetic patients, vascular endothelial cells are commonly damaged by oxidative stress that ultimately contributes to the development of macrovascular and microvascular diseases. Our study also found that SOD and GSH-Px activity in the DM + CI group were decreased, and the MDA content increased, which suggested the role of the oxidative stress of the peripheral lymphocytes in DM patients complicated with CI.

Multiple mechanisms are involved in the pathogenesis of ischemic stroke, of which oxidative stress injury and apoptosis seem to be the most critical events [6]. More and more research has shown that oxidative stress is a significant mechanism leading to brain tissue injury after cerebral ischemic stroke [24]. The present study showed that the SOD and GSH-Px activities were decreased in the CI group, and the MDA content was increased. It lasted for a month, and in the third month, the results returned to normal. Our findings are in agreement with previous studies as it has shown that oxidative stress is a significant mechanism leading to brain tissue injury after cerebral ischemic stroke [24].

In addition, this study found that SOD and GSH-Px activities of DM patients complicated with CI were lower than those in the DM group and CI group, suggesting a stronger oxidative stress response of peripheral blood lymphocytes in DM patients complicated with CI. These results suggested oxidative stress plays a role in diabetes and diabetes complications, and the regulation of oxidative stress may be a therapeutic approach for the treatment and prevention of diabetic complications.

GSK-3 is a protein kinase that is involved in multiple signaling pathways, including those that regulate GS metabolism, insulin sensitivity, Wnt, and TGF-β signaling [9,25]. GSK-3 exists in two isoforms in mammals: GSK-3α and GSK-3β. GSK3β is more widely expressed throughout different brain regions. It is involved in various cellular processes, including insulin signaling, glycogen metabolism, and neuronal apoptosis. In recent years, studies have found that PI3K/Akt/GSK-3β is a key insulin signaling pathway mainly regulated by upstream factors like IGF-1 and external stimuli [26,27]. In the presence of insulin stimulation, the PI3K-Akt signaling pathway is initiated, inducing the phosphorylation of GSK3, thereby stimulating glycogen synthesis [10]. This study confirmed that phosphorylation of GSK-3β in peripheral lymphocytes of diabetic patients was decreased, which suggested the increase of GSK-3β activity. The result was consistent with previous studies [28]. Due to the critical role of GSK-3 in insulin signaling and GS metabolism, GSK-3 has emerged as a very promising therapeutic target for the treatment of type 2 diabetes.

It is generally recognized that T2DM-associated vascular disease is mainly associated with vascular endothelial injury and endothelial dysfunction, which are the initial steps leading to atherosclerosis [23]. There have been studies shown that damage to the cerebral vascular endothelium activated the PI3K-Akt pathway, led to decreased GSK-3 activity, and promoted smooth muscle cell proliferation and vascular remodeling [1,29]. In the present study, phosphorylation of GSK-3β in DM patients complicated with CI was decreased. The results were similar to those observed in animal experiments [28]. In this study, GSK-3β activity was found in DM patients complicated with CI, confirming that the mechanism of diabetes causing CI may be achieved by affecting the GSK-3β activity.

Studies on cerebral ischemic stroke have found that the PI3K/Akt signaling pathway can promote cell survival, inhibit cell apoptosis, and play an important role in neuroprotection during cerebral ischemia-reperfusion [30]. It has been suggested that the PI3K-Akt pathway is the upstream signal pathway that negatively regulates the expression of GSK-3β. GSK-3β is inactivated by the activation of PI3K/AKT, while AKT protects the brain by promoting angiogenesis, neurogenesis, anti-apoptosis, and anti-inflammation. Activation of the AKT/GSK-3βsignaling pathway enhances the neurovascular recovery of in response to ischemic brain injury [31]. In this study, phosphorylation of GSK-3β in patients with CI was lower, which means that the damage of the cerebral vascular was more serious. In this study, phosphorylation-GSK-3β in patients with CI decreased from day 1 to 1 month after the disease and returned to normal on the third month. This indicates that GSK-3β activity was enhanced. The results confirmed the role of GSK-3β in the pathogenesis of CI.

Because of the association between oxidative stress and diabetes, multiple attempts have been made to treat DM patients with antioxidant supplements, such as enzymatic antioxidants like mimics, vitamin C, and vitamin E [2,32]. However, these therapeutic approaches have been unsatisfactory. Therefore, there is a trend for searching for new drugs that can target novel pathways involved in the pathogenesis of diabetes. Regulation of GSK-3 was found to have therapeutic effects in cancer, nervous system, and diabetic diseases. GSK-3 is a negative regulator of insulin signaling, and Gsk-3 was found to be activated immediately after the initiation of the oxidative stress pathway in stroke [31,33]. It has been found that the inhibition of Gsk-3 shows neuroprotection via reducing oxidative stress and inflammation in cerebral ischemia/reperfusion [6]. In our study, the results showed that GSK-3 activity was increased in the lymphocytes of DM patients complicated with CI. Thus, it is promising to design specific GSK-3 inhibitors as future drugs for the treatment and prevention of diabetes and complications.

DM is a group of metabolic diseases characterized by hyperglycemia, resulting from defects in insulin production and/or insulin action and impaired carbohydrate, lipid, and protein metabolism [34]. This study showed that TC, and LDL-C were all higher in the DM group compared with the control group. In fact, diabetic patients often exhibit an atherogenic pattern that includes higher levels of TC, LDL-C, and TGs and lower levels of HDL-C than those who do not develop diabetes [35]. Accumulation of multiple risk factors such as hypertension, dyslipidemia, hyperinsulinemia, and hyperglycemia leads to cerebrovascular and cardiovascular complications in diabetic patients. In the DM + CI group, lipids were higher than in the control group, and LDL-C was higher than in both DM group and CI group. All results showed that disorders of lipid metabolism were more obvious in DM patients complicated with CI. This study suggested that blood pressure in the DM + CI group was found to be higher than that in the control group. Patients with DM are in a state of hyperglycemia for a long time, and blood lipids and GS levels are increased significantly, resulting in cerebral vasculopathy and hemodynamic abnormalities, leading to atherosclerosis, and then developing CI [36,37]. DM patients complicated with CI were found to have abnormal blood GS, blood pressure, and lipids, but more studies should be carried out on the correlation between blood pressure, blood lipids, and the incidence of DM complicated with CI.

Oxidative stress is a major upstream event for diabetes complications as well as insulin resistance development, inducing pathophysiologic molecular mechanisms and initiating a cascade of deleterious pathways leading to insulin resistance and DM [38]. Oxidative stress can result in impairment of insulin signal transduction via downregulation of proteins involved in the normal IST such as Akt, IRS, IRS-1, and GSK-3. They are downregulated by oxidative stress, thereby impairing insulin sensitivity, leading to insulin resistance and DM. Balbaa et al. determined IST elements in the brain of diabetic rats, and they found that oxidative stress markedly reduced IST element expression as p-IRS, p-AKT, and GSK-3β in brain tissues [39]. In diabetic patients, vascular endothelial cells are usually damaged by oxidative stress, which ultimately leads to the development of macrovascular and microvascular disease. There have been studies shown that damage to the cerebral vascular endothelium activated the PI3K-Akt pathway, led to decreased GSK-3 activity, and promoted smooth muscle cell proliferation and vascular remodeling [1,29]. The repressed Akt and stimulated GSK-3β have been proven to exacerbate neuronal damage in diabetic encephalopathy. In diabetes, GSK-3 is an important target of insulin signaling, and its phosphorylation at specific residues leads to the inhibition of GSK-3 activity. Increased GSK-3 activity has been observed in T2DM patients and mice with diabetes, indicating its involvement in impaired insulin signaling [40]. Due to the critical role of GSK-3 in insulin signaling and GS metabolism, GSK-3 has emerged as a very promising therapeutic target for the treatment of type 2 diabetes.

Overall, targeting GSK-3 is a promising therapeutic target for the treatment of type 2 diabetes. However, more research is needed to fully understand the role of GSK-3 in type 2 diabetes and to develop selective inhibitors involved in the disease.

## 5 Conclusions

The present study demonstrated that the oxidative stress in peripheral lymphocytes of DM patients complicated with CI was increased, and the GSK 3 activity was enhanced. The results suggested the role of oxidative stress on the DM and its complications. Also, the increased GSK-3β activity, causing more severe cellular damage, may be one of the mechanisms by which oxidative stress causes DM complicated with CI. Overall, targeting GSK-3 is a promising therapeutic target for the treatment of type 2 diabetes. These results proved that oxidative stress regulates changes in GSK-3 activity and may have a role in diabetes and its complications, and inhibitors targeting oxidative stress and GSK-3 may be relatively promising therapeutic measures. However, more research is needed to fully understand the role of GSK-3 and its isoforms in type 2 diabetes and to develop selective inhibitors that target only the specific isoform involved in the disease.

In addition, the study also suggested that DM patients with CI had higher blood pressure, blood GS and blood lipids, especially LDL cholesterol. It provided that well controlling of LDL cholesterol may reduce the incidence of DM with CI. Hence, further researches are necessary to explore the relationship between them, like adding other analaytic methods such as single correlation or multiple regression analyses.
